# *In Vivo* Molecular Optical Coherence Tomography of Lymphatic Vessel Endothelial Hyaluronan Receptors

**DOI:** 10.1038/s41598-017-01172-x

**Published:** 2017-04-24

**Authors:** Peng Si, Debasish Sen, Rebecca Dutta, Siavash Yousefi, Roopa Dalal, Yonatan Winetraub, Orly Liba, Adam de la Zerda

**Affiliations:** 1 Molecular Imaging Program at Stanford, Stanford, California 94305 USA; 2 Department of Structural Biology, 299 Campus Drive West, Stanford, California 94305 USA; 3 Department of Radiation Oncology, 875 Blake Wilbur Drive, Stanford, California 94305 USA; 4 Department of Ophthalmology, 2452 Watson Ct, Stanford, California 94303 USA; 5 Department of Electrical Engineering, 350 Serra Mall, Stanford, California 94305 USA; 6 Bio-X Program, Stanford University, Stanford, California 94305 USA

## Abstract

Optical Coherence Tomography (OCT) imaging of living subjects offers increased depth of penetration while maintaining high spatial resolution when compared to other optical microscopy techniques. However, since most protein biomarkers do not exhibit inherent contrast detectable by OCT, exogenous contrast agents must be employed for imaging specific cellular biomarkers of interest. While a number of OCT contrast agents have been previously studied, demonstrations of molecular targeting with such agents in live animals have been historically challenging and notably limited in success. Here we demonstrate for the first time that microbeads (µBs) can be used as contrast agents to target cellular biomarkers in lymphatic vessels and can be detected by OCT using a phase variance algorithm. This molecular OCT method enables *in vivo* imaging of the expression profiles of lymphatic vessel endothelial hyaluronan receptor 1 (LYVE-1), a biomarker that plays crucial roles in inflammation and tumor metastasis. *In vivo* OCT imaging of LVYE-1 showed that the biomarker was significantly down-regulated during inflammation induced by acute contact hypersensitivity (CHS). Our work demonstrated a powerful molecular imaging tool that can be used for high resolution studies of lymphatic function and dynamics in models of inflammation, tumor development, and other lymphatic diseases.

## Introduction

Molecular imaging techniques provide powerful approaches for visualizing biological activities in living subjects; however, current molecular imaging modalities must compromise either imaging depth of penetration or spatial resolution^1^. To date, no molecular imaging modality is capable of achieving millimeter-scale depth of penetration while maintaining cellular-level resolution *in vivo*. Such a technology, if available, would enable the recording of molecular information from hundreds of millions of cells in whole living tissues. This is the focus of the present work.

Optical Coherence Tomography (OCT) is a non-invasive imaging technique that offers millimeters depth of penetration into tissue while maintaining high spatial resolution (1–10 µm) throughout the imaging depth^2, 3^. Moreover, OCT produces three-dimensional (3D) images with high temporal resolution, reaching acquisition speeds up to 10^5^~10^6^ A-scan depth-lines per second^4^. Through the detection of backscattered light, OCT provides images of tissue microstructures such as epidermis, dermis, cartilage, and lymphatic vessels with near-histological detail. OCT is also sensitive to phase changes that arise from minute movements of scatterers during the acquisition of consecutive scans. This sensitivity enables a phase variance-OCT (PV-OCT) mode, which can be used to detect the presence of blood vessels due to the motion of red blood cells^5–8^.

Despite favorable tissue penetration and spatial resolution, OCT typically only conveys structural information about biological samples since most molecular biomarkers do not produce unique OCT signals. For this reason, OCT-detectable exogenous contrast agents are highly desirable (and arguably vital) to achieve molecular OCT imaging. Several contrast agents have been proposed in the past for various extensions of OCT such as magnetic nanoparticles for magneto-motive OCT^9, 10^, methylene blue for pump-probe OCT^11^, and gold nanoparticles for photothermal OCT^12–15^. However, these and other agents have experienced limited success for targeted biomarker imaging in living subjects, most commonly due to the limited contrast agent sensitivity and tissue background signal.

Here we report the first demonstration of true targeted molecular imaging using OCT. We used polystyrene-based µBs as OCT contrast agents due to their high scattering cross sections and large refractive index mismatch with tissue. We addressed the challenges of targeting microparticles to small biomolecules by devising a pre-targeting approach. In this design, biotinylated antibodies against a specific lymphatic biomarker LYVE-1 were initially injected, followed by the administration of streptavidin-coated µBs (stp-µBs). The pre-targeting approach successfully labeled µBs to the LYVE-1 receptors expressed on lymphatic endothelial cells by utilizing the high affinity of streptavidin for biotin, which is several orders of magnitude higher than that of an antibody. LYVE-1-bound µBs were then detected by a phase variance-based algorithm we developed to achieve ultrahigh (single-µB) contrast sensitivity. The accuracy of the contrast agent detection algorithm was validated in phantoms and in living mouse lymphatic capillaries, respectively. We used this experimental approach to study active targeting pattern of µBs to LYVE-1 receptors, showing that nontargeted µBs were cleared within 90 min of administration and targeted µBs exhibited clear contrast to the tissue. By using our prototype molecular OCT method, we report the first *in vivo* observation of LYVE-1 down-regulation during contact hypersensitivity (CHS) induced acute skin inflammation in a murine model. This demonstration shows the biological practicality of the presented molecular OCT method and lays a foundation for future translational research.

## Results

### Characterizations of µB contrast agents

**Figure 1:**
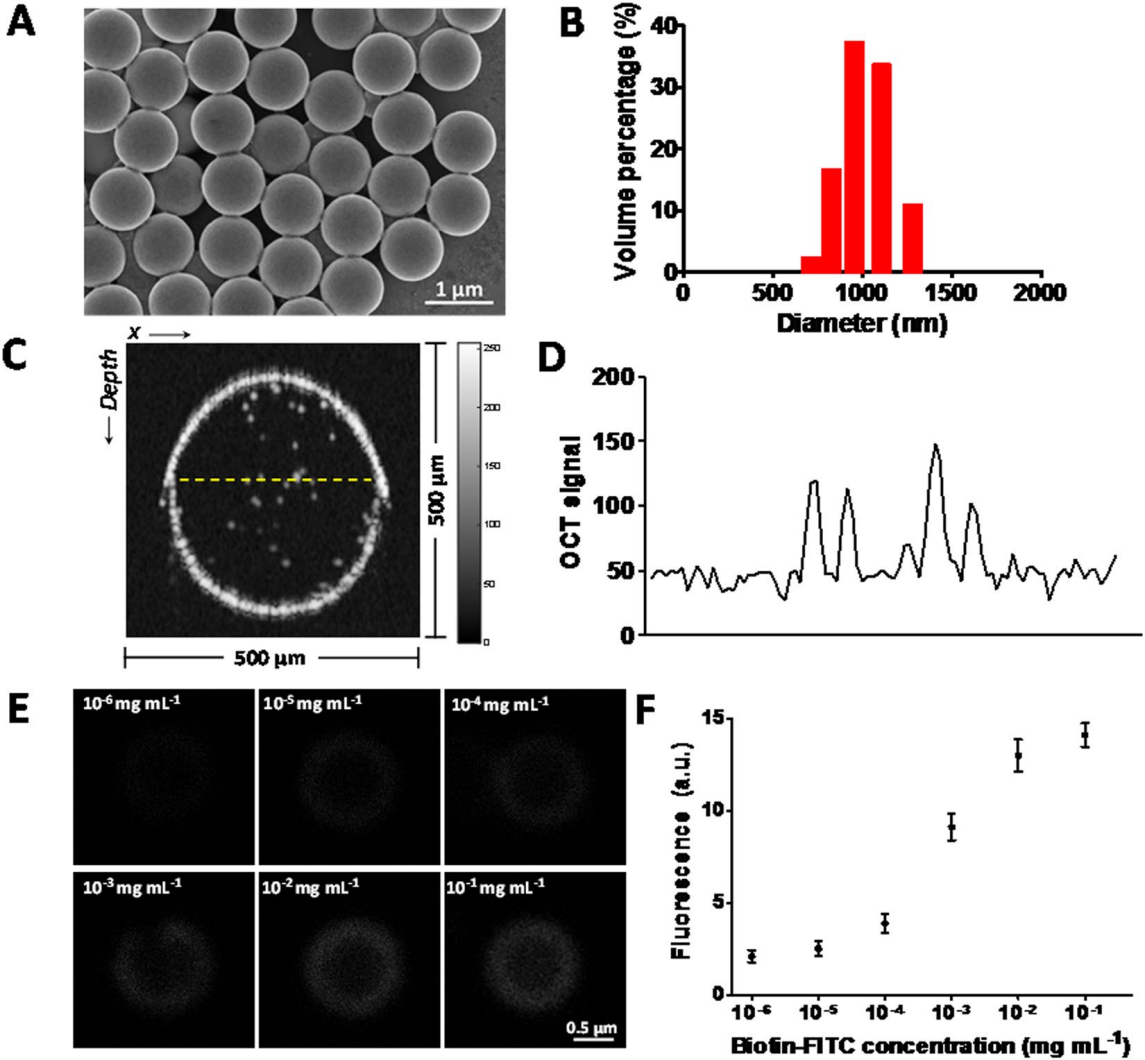
Characterizations of stp-µB contrast agents. (**A**) SEM micrograph of stp-µBs. (**B**) Representative size distribution of stp-µBs as measured by dynamic light scattering (DLS). (**C**) OCT B-scan image showing the scattering signals of stp-µBs in water in a glass capillary tube of 400 μm inner diameter. (**D**) OCT intensity signal profile along the yellow dashed line marked across the capillary tube in (**C**). (**E**) Confocal fluorescent images of the central plane of a single FITC-µB obtained from a titration assay, in which 10 mg mL^−1^ stp-µBs were reacted with different concentrations of biotin-FITC (from upper left to lower right: 10^−6^, 10^−5^, 10^−4^, 10^−3^, 10^−2^, 10^−1^ mg mL^−1^). (**F**) The relationship between the mean fluorescent intensity of the FITC-µBs and the corresponding biotin-FITC concentration in the titration assay. Error bars depict the SEM of FITC-µB fluorescence intensities at each concentration.

The size distribution and dispersity of the µB contrast agents were investigated by scanning electron microscopy (SEM) and dynamic light scattering (DLS). SEM revealed that the stp-µBs have very uniform spherical morphologies with relatively smooth surfaces (Fig. 1A). DLS measurement showed the particles have an average diameter of 1.01 μm with a narrow size distribution (Fig. 1B), indicative of high monodispersity. µBs possess a large geometric scattering cross-section (3.23 µm^2^ per µB) and a large refractive index mismatch relative to water (1.59 to 1.33), which led to strong scattering and thus high OCT signal, especially in low background environments (Fig. 1C and D). This enabled contrast agent sensitivity that approaches single particle detection capabilities. In order to verify the biotin binding capacity of the stp-µB, we conducted a titration assay by reacting stp-µBs with a gradient of biotin-FITC molecule concentrations and measuring the fluorescence intensity of the resulting dye-conjugated particles. Minimal fluorescence was detected from the central plane of each bead for low biotin-FITC concentrations (i.e., 10^−6^ and 10^−5^ mg mL^−1^), whereas stronger fluorescent signals from the bead was observed with further increase of the biotin-FITC concentrations (Fig. 1E and Supplementary Fig. S1). Fluorescent intensity reached saturating levels at biotin-FITC concentrations higher than 10^−2^ mg mL^−1^ (Fig. 1F). We thus calculated that the average binding capacity of each stp-µB was 1.8 × 10^5^ biotin molecules and the density of active streptavidin molecules on the surface of each bead was 1.4 × 10^4^ μm^−2^. Due to the high density of active streptavidin and high biotin-binding capacity, the stp-µBs are suitable candidates for active targeting when used alongside biotinylated pre-targeting antibodies.

### Phantom study for PV-OCT detection of targeted µBs

**Figure 2:**
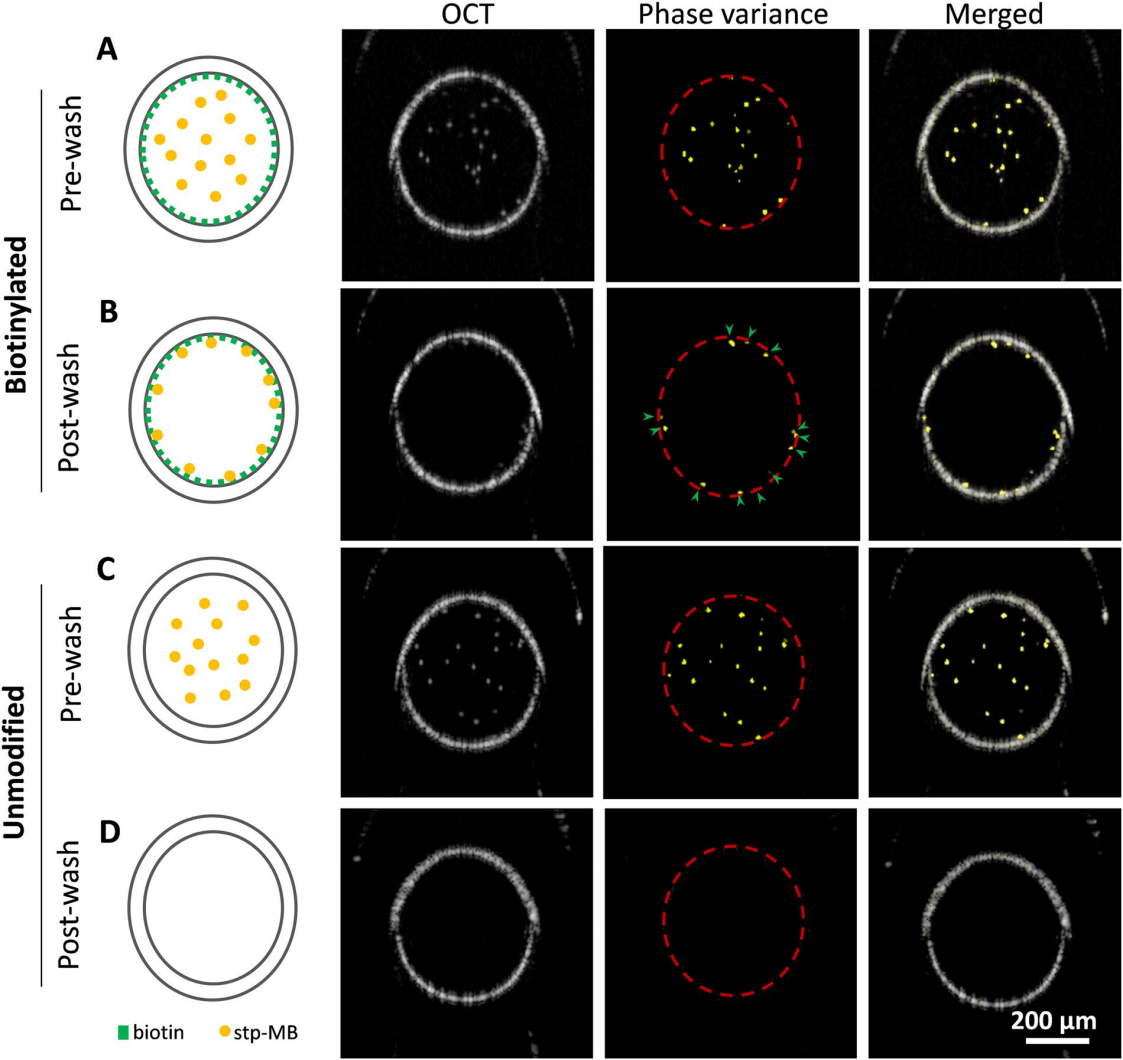
OCT scattering and phase variance signals of flowing and target-bound µBs in biotinylated (**A**,**B**) and unmodified (**C**,**D**) capillary tubes before (**A**,**C**) and after (**B**,**D**) wash. Columns from left to right: (1) Illustrations of the capillary tube modification and distributions of stp-µBs inside the capillary tubes, (2) OCT B-scan images showing the OCT scattering signals of stp-µBs and the reflections from the capillary tube glass/water interfaces, (3) detected phase variance signals (the glass/water interfaces are marked by red dashed lines) and (4) Superimposition of the detected phase variance signals on ﻿the OCT B-scan images.

We used a capillary tube as a phantom to verify the ability to detect µB contrast agents attached to molecular targets using an OCT phase variance algorithm. Contrast agents were identified based on phase variance of their scattered light instead of their OCT scattering signals, since the tissue produces strong OCT scattering signals bu﻿t little phase variance. In the phantom setup, biotin-functionalized capillary tubes were used to capture stp-µBs. Unmodified capillary tubes were used as controls to demonstrate µB binding specificity. Actively-flowing µBs produced strong OCT intensity and their movements were detectable using the phase variance algorithm (Fig. 2A and C). The detected phase variance signals registered well with their respective OCT signals, reflecting the accuracy of the detection when the beads were traveling through the capillary tube (a mimic of vessel structure). After washing, no OCT signals were observed in the free space of either biotinylated or unmodified capillary tubes (Fig. 2B and D). However, phase variance signals were sti﻿ll detected at multiple locations on the inner surface of the biotinylated capillary tube (arrows in Fig. 2B). These signals presumably originated from sub-pixel displacements of µBs after binding to the capillary tube via biotin-streptavidin reaction. T﻿hese sub-pixel movements produced phase changes of the backscattered light collected during the imaging interval. Although punctate OCT scattering signals can also be observed on the capillary tube inner surface (Fig. 2B), it is difficult to distinguish these signals from the inherent strong reflections at the water/capillary tube interface. The co-localization of these OCT scattering signals with the detected phase variance signals indicate our imaging algorithm is capable of detecting µBs that were bound to their molecular targets in a accu﻿rate manner. Neither phase variance nor OCT signals could be seen inside the unmodified capillary tube after washing (Fig. 2D), indicating there was no non-specific binding; all unbound µBs were cleared from the capillary tube after the washing step.

### Validation of the OCT contrast agent signals *in vivo*

**Figure 3:**
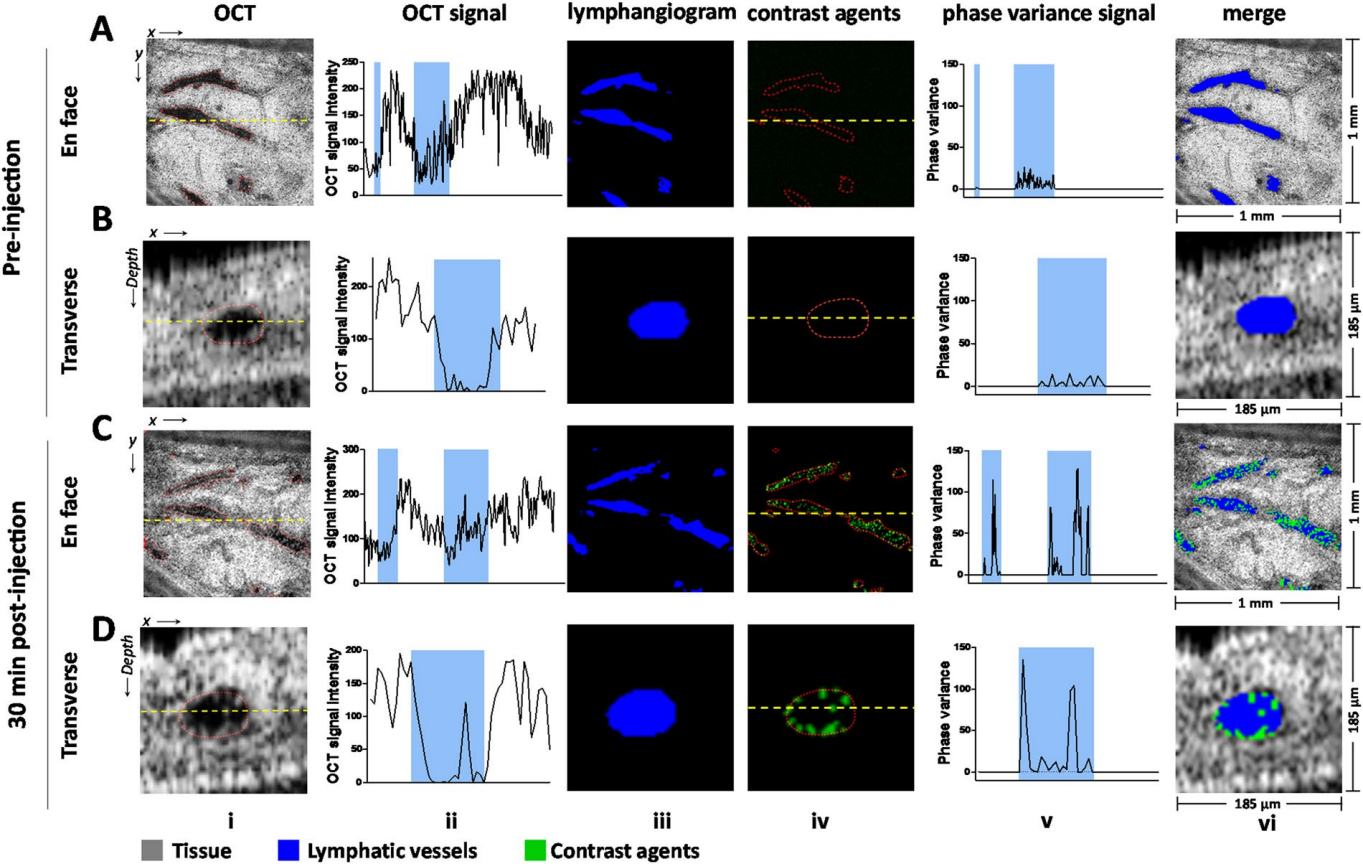
Validation of the phase variance contrast signals *in vivo*. Image columns from left to right: (i) the *en face* (**A** and **C**) and transverse (**B** and **D**) OCT microstructure images, (ii) the corresponding OCT signals along the yellow dashed line marked on the OCT microstructure images, (iii) the segmented lymphatic vessels, (iv) the contrast agent signals detected by﻿ phase variance, (v) the corresponding phase variance signals along the yellow dashed line marked across the contrast agent signals, and (vi) merged images of OCT microstructure, lymphatic vessels and lymphatic contrast signals at pre-injection (**A** and **B**) and 30 min post-injection (**C** and **D**). The red dashed line on the OCT microstructure and lymphatic contrast images indicate the outline of the segmented lymphatic vessels. The blue windows on the OCT signal and phase variance signal graphs indicate the locations of the lymphatic vessels.

Since lymphatic fluids are almost optically transparent^16–18^, the lymphatic vessels in the mouse pinna exhibit very low OCT signal intensities that are close to the thermal noise of the imaging system (Fig. 3A,B, Columns i and ii). Owing to this transparency, lymphatic vessel networks can be identified in OCT microstructure images using automatic segmentation algorithms^19, 20^ (Fig. 3A–D, Column iii). Prior to contrast agent injection, only noise-level endogenous phase variance signals were present in the segmented lymphatic vessels (Fig. 3A,B, Column v). This baseline noise was removed after setting an appropriate phase variance threshold signal, thus no contrast agent signals were present inside the lymphatic vessels at pre-injection (Fig. 3A,B, Columns iv and vi). OCT signals corresponding to µBs can be observed clearly in the lymphatic vessels from both the *en face* and transverse OCT microstructure images (Fig. 3C,D, Column i) 30 minutes after the subcutaneous (s.c.) injection of µBs. However, when the µBs were very close to the lymphatic endothelium tissue, their OCT signals were mixed with the OCT signals of the surrounding tissue, which precludes high-specificity µB detection (Fig. 3C,D, Column ii). Similar to capillary experiments, PV-OCT resolved the µBs from the high scattering of the neighboring lymphatic endothelium. Phase variance signals were detected both inside the free space of the lymphatic lumen and adjacent to the lymphatic endothelium (Fig. 3C,D, Column v), corresponding to unbound flowing and putative LYVE-1-targeted µBs, respectively. The detection of µB correlated phase variance signals enabled us to image and track the contrast agents in the lymphatic vessels *in vivo* (Fig. 3C,D, Columns iv and vi).

### Longitudinal imaging of LYVE-1 targeted µBs *in vivo*

**Figure 4:**
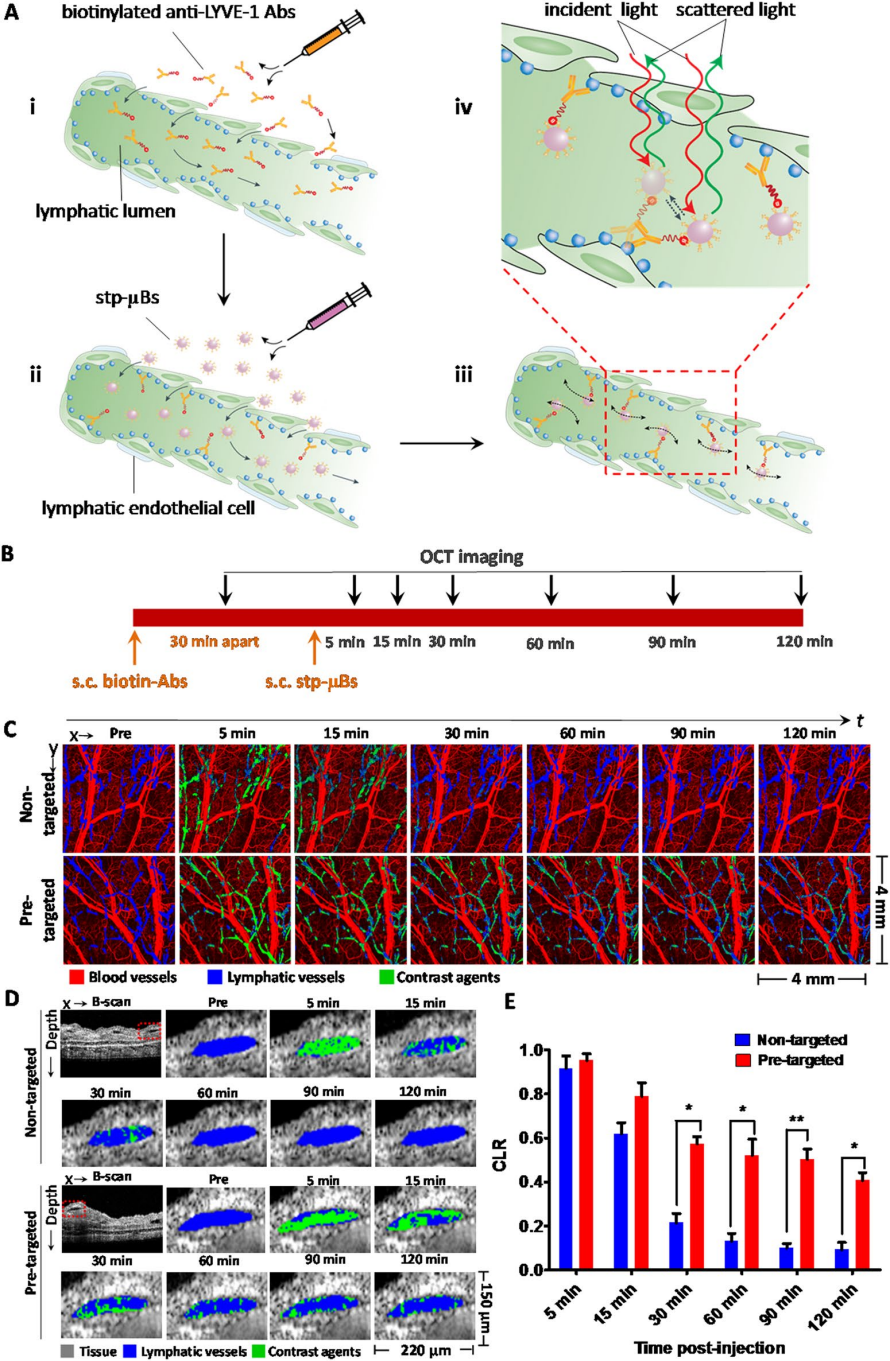
Longitudinal imaging of LYVE-1 targeted µB *in vivo*. (**A**) Schematic illustration of LYVE-1 targeting mechanism achieved by the pre-targeting strategy and the PV-OCT method for contrast agent detection. (**B**) The timeline of subcutaneous (s.c.) injections of the contrast agents (orange arrows) and longitudinal OCT imaging time points (black arrows). (**C**) Time-lapse *en face* merged projection images showing angiogram (red), lymphangiogram (blue), and lymphatic contrast agent signals (green) in the ear tissues of non-targeted and pre-targeted groups of mice before and at various time points following s.c. injection. (**D**) Time-lapse transverse merged images displaying the tissue microstructure (gray), lymphangion (blue), and the contrast agent signals (green) in the ears of non-targeted and pre-targeted groups of mice at pre-injection and various time points post-injection. (**E**) The contrast-to-lymphatic ratios of the non-targeted versus the pre-targeted groups of mice at different imaging time points post-injection. Error bars represent ± SEM (n = 4 mice). *P < 0.05, **P < 0.001.

We devised a pre-targeting strategy to deliver the LYVE-1 targeted contrast agents to mouse lymphatic vessels. As illustrated in Fig. 4Ai, *in vivo* imaging experiments began with s.c. administration of biotinylated anti-LYVE-1 antibodies in the ears followed by the injection of stp-µBs after 30 minutes in the pre-targeted group of mice (n = 4). The initial injection of biotinylated anti-LYVE-1 antibodies enables them to bind to their respective molecular targets, and the subsequent injection of stp-µBs allows the labeling of contrast agents to the pre-targeted antibodies via biotin-streptavidin interaction (Fig. 4Aii). Since the streptavidin has a much higher binding affinity for biotin than that of the antibodies for biomarkers, this design allows the micro-sized contrast agent to bind strongly to their molecular targets, possibly with the added benefits of avidity effects. Upon binding to the LYVE-1 receptors, the µBs continue to move slightly in place due to the flexibility of the antibody and biotin linkers (Fig. 4Aiii). These slight *in situ* movements yield the phase variance that can be leveraged to detect the biomarker-bound contrast agents (Fig. 4Aiv). In order to characterize the clearance pattern of the unbound µBs and the specificity of the pre-targeting strategy, a control experiment was designed by injecting biotinylated isotype control antibodies followed by stp-µBs in non-targeted group of mice (n = 4). 3D volume images of the mice pinna were acquired at 15 min post-antibody injection as a baseline, post-injection images were taken at 5 min, 15 min, 30 min, 60 min, 90 min and 120 min after stp-µB administration (Fig. 4B). The longitudinal imaging allowed us to observe the clearance timeline of the stp-µBs in the lymphatic vessels and determine the optimal post-injection time for imaging the targeted contrast agents.

We first compared the *en face* ear images of non-targeted and pre-targeted groups of mice in a longitudinal manner (Fig. 4C). Acquired *en face* images displayed co-localized angiograms, lymphangiograms, and contrast agent signals obtained by projecting segmented 3D data volumes of blood vessels, lymphatic vessels and contrast agent signals on 2D (x-y) planes (Supplementary Fig. S2). Then we longitudinally compared the transverse images (Fig. 4D), which were obtained by co-localizing the OCT B-scan images, segmented lymphatic vessel images, and contrast agent signal images in the same x-z plane (Supplementary Fig. S3). The *en face* and transverse images showed no contrast agent signals in the lymphatic vessels prior to stp-µB administration. Abundant contrast agent signals were identified in the lymphatic lumen of both pre-targeted and control mice after 5 min of injection, indicating the drainage of the injected stp-µBs within lymphatic capillaries. Greater reduction of the contrast agent signals was observed in the non-targeted than in the pre-targeted group of mice at 15, 30 and 60 min post-injection. After 90 min of injection, quite a few contrast agent signals persisted in the lymphatic vessels of the pre-targeted mice (Fig. 4C and Supplementary Movie S1). Notably, these signals were distributed alongside the lymphatic endothelium (Fig. 4D and Supplementary Fig. S4). By comparison, very few contrast agent signals can be spotted in the lymphatic vessels of the non-targeted mice ears at the corresponding imaging time points (Fig. 4C,D and Supplementary Movie S2). These results suggested that the unbound stp-µBs can be effectively cleared by the lymphatic fluid within 90 min of injection and that the observed contrast agent signals in the pre-targeted mice resulted specifically from LYVE-1 targeted µBs. 3D images show that each single target-bound stp-µB can be clearly observed on the inner surface of lymph endothelium at 90 min post-injection (Supplementary Movie S3), indicating ultrahigh contrast agent sensitivity. The results also suggest there is little non-specific adhesion of either biotinylated antibodies or stp-µBs on the lymphatic endothelium, and the pre-targeting approach has very high specificity towards imaging LYVE-1 biomarkers *in vivo* with the molecular OCT method.

To further validate the targeting specificity of this approach, a competition assay was conducted by injecting a mixture of free non-biotinylated anti-LYVE1 and biotinylated anti-LYVE1 antibodies with a ratio of 10:1 followed the injection of stp-µBs. The observed mice ear lymphatic vessels showed similar clearance patterns to the non-targeted group (Fig. S5). The lymphatic vessels were filled with stp-µBs at 5 min post-injection, and contrast agent signals decreased dramatically at 15 and 30 min post-injection. Only sparse contrast can be observed in the lymphangion after 30 min post-injection. This result further corroborates that there is little non-specific absorption of the biotinylated anti-LYVE-1 or stp-µBs on the surface of lymphatic endothelium tissue.

**Figure 5:**
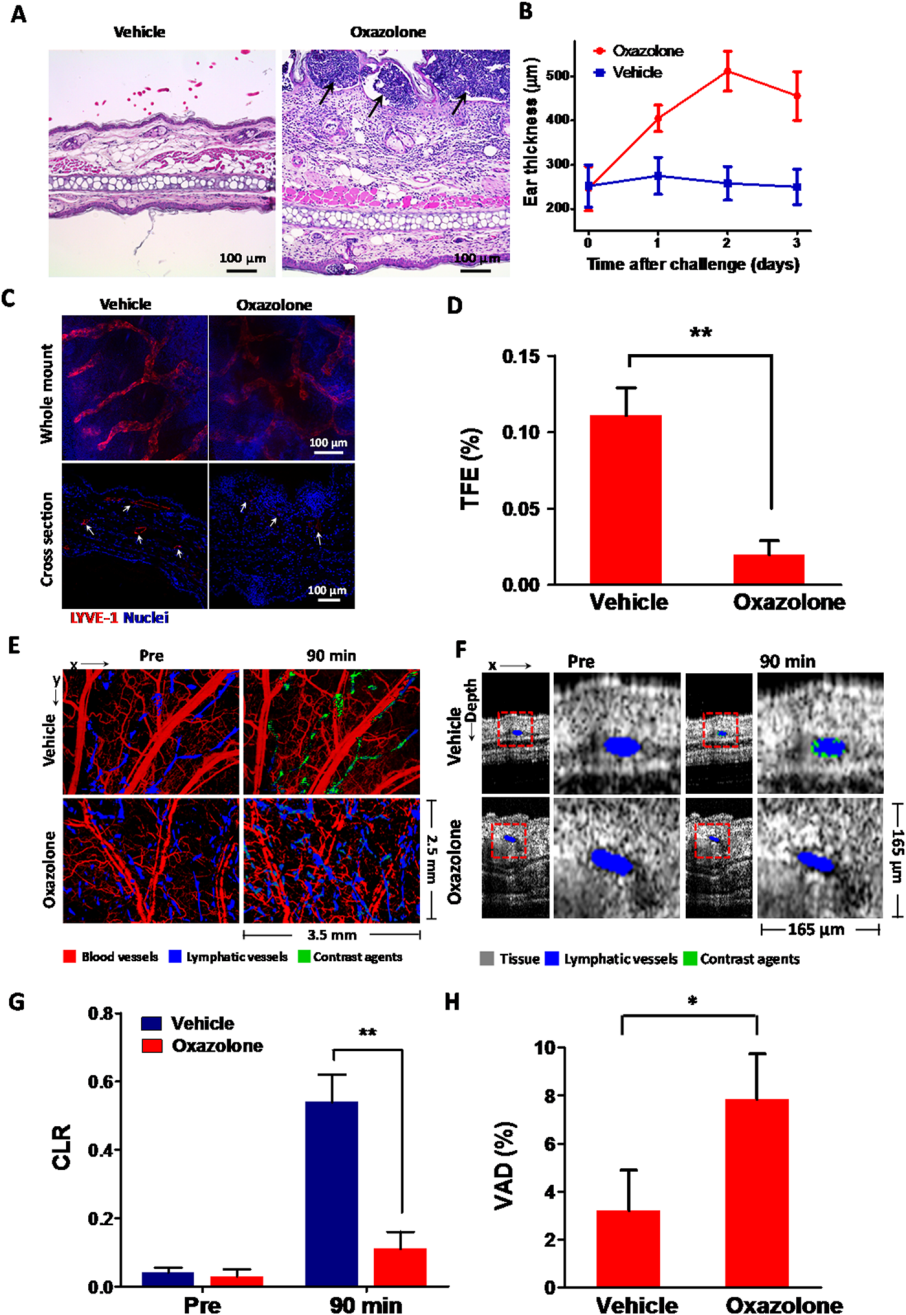
LYVE-1 imaging with CHS induced inflammation mouse models. (**A**) The hematoxylin and eosin (H&E) staining of control and inflamed mice ears after two days of OXA treatment. Arrows indicate the locations of intraepidermal abscesses. (**B**) The thickness of the vehicle- and OXA-treated mice ears monitored at different days post-challenge. (**C**) Representative fluorescent microscopy images show the immunohistochemical (IHC) staining of LYVE-1 (Alexa 594-anti-LYVE-1, red) and nuclei (DAPI, blue) in whole-mount and cross-sectional tissue sections of control and inflamed mice ears after 2 days of OXA treatment. The paired fluorescent images of vehicle- and OXA-treated ear tissues were captured by confocal microscopy with identical illumination and acquisition parameters. The whole-mount fluorescent images were obtained as maximum intensity projections of 50 confocal images scanned over a tissue depth of 100 μm. Arrows in the cross-sectional images indicate the IHC staining of LYVE-1 along the lymphatic endothelium. (**D**) Total fluorescence efficiency (TFE) of LYVE-1 in the IHC whole mount images. Data pooled over 1000 images of tissue sections from 3 vehicle-treated and 3 OXA-treated mice. **P = 0.00074 (unpaired *t*-test). (**E**) *En face* projection images of angiogram (red), lymphangiogram (blue), and lymphatic contrast (green) signals of vehicle- and OXA-treated mice pinnae at pre-injection and 90 min post-injection of contrast agents. (**F**) Transverse images of ear tissue microstructure (gray), lymphangion (blue), and lymphatic contrast agents (green) of vehicle- and OXA-treated mice pinna at pre-injection and 90 min post-injection of contrast agents. (**G**) CLRs of vehicle- and OXA-treated ears at pre-injection and 90 min post-injection after 2 days of OXA challenge. Bars show means ± SEM, n = 4 mice; **P = 0.00091. (**H**) Lymphatic vessel area density of vehicle- and OXA-treated mice ears at 2 days post-challenge. Bars show means ± SEM (n = 4 mice). *P = 0.0046.

In order to quantitatively and statistically compare the levels of contrast agent signals in non-targeted and pre-targeted mice ears at different longitudinal imaging time points, contrast-to-lymphatic signals ratios (CLRs) were determined. The CLR was calculated by dividing the total pixel area count of the contrast agent signals by the total positive pixel area count of the lymphaangiogram. As shown in Fig. 5E, non-targeted and pre-targeted mice show very close CLRs at 5 min post-injection. Although the CLRs of both groups decline at 15 min post-injection, the pre-targeted group shows a 27% higher CLR than its counterpart. From 30 min post-injection onward, the CLR of the pre-targeted mice become significantly higher than that of the non-targeted controls. The pre-targeted group shows 2.7-, 4.0-, 5.1- and 4.4-fold higher CLRs than the control group at 30 min, 60 min, 90 min and 120 min post-injection, respectively. Statistical analysis shows the difference of CLRs between the two imaging groups become most significant (*P* = 0.0006, unpaired *t*-test) at 90 min post-injection. We also compared the CLRs of the competitive assay group and pre-targeted group. The CLRs of the pre-targeted group of mice are significantly higher than the competitive assay group from 30 min post-injection onwards (Fig. S6). Statistical analysis confirms the high specificity of the pre-targeting approach for molecular imaging lymphatic biomarkers with µB-enhanced PT-OCT and suggests that 90 min post-injection is the optimal time point for imaging LYVE-1 targeted contrast agents.

### *In vivo* imaging inflammation induced LYVE-1 down-regulation

To demonstrate the preclinical utility of µB-enhanced molecular OCT, we applied this imaging approach to study the influence of inflammation on LYVE-1 expression *in vivo*. The inflammation of mice ear tissues was induced by acute CHS, which was generated by sensitizing the mice on their abdomen with oxazolone (OXA) followed by challenging their ears with OXA 5 days after the sensitization. As a control, only vehicles were applied to abdomen and ears. Two days after the challenge, the ears were collected for histological analysis (Fig. 5A). Compared to the vehicle-treated ear, the OXA-treated counterpart showed significant swelling and expansion of the dermis. This swelling was caused by the infiltration of the inflammatory cells such as neutrophils, macrophages and lymphocytes. Inflammation was evident by neutrophil accumulation (intraepidermal abscesses) in multiple areas of the superficial epidermis (Fig. 5A, black arrows) and various degrees of hyperplasia of the surrounding epidermis. The thickness of vehicle- and OXA-treated mice was measured daily after the challenge (Fig. 5B). It is observed that the mice pinna exhibited an average thickness of ~250 μm prior to treatment. The thickness of vehicle-treated mice ears increased slightly to 275 μm after one day of treatment and finally decreased to 250 μm by Day 3 post-treatment. By contrast, the ear thickness of OXA-treated mice increased substantially to 405 μm in the first day after challenge and reached a maximum thickness of 512 μm at Day 2 post-challenge. The challenged ear showed a slight decrease in thickness to 456 μm in the third day after treatment, likely due to natural relief.

Next, we examined the expression of LYVE-1 in vehicle- and OXA-treated mice pinnae 48 hours post-treatment by comparative immunostaining (Fig. 5C and Supplementary Fig. S7). In whole-mount samples of vehicle-treated ears, LYVE-1 staining showed strong fluorescence across the branched network of blind-ended lymphatic vessels, indicating high levels of constitutive LYVE-1 expression on lymphatic endothelial cells (LECs). In contrast, markedly reduced levels of LYVE-1 staining were observed in contralateral inflamed tissue. In cross-section tissue slices, distinct LYVE-1 staining was visible within the lymphatic endothelium of the vehicle-treated ears, whereas minimal fluorescence of LYVE-1 staining can be observed in OXA-treated ears suffering from inflammation. These results indicated the LYVE-1 expression was significantly down-regulated after the OXA-challenge, which is consistent with previous reports modeling CHS^21^. We quantitatively assessed 50 vehicle- and OXA-challenged ear samples by immunohistochemistry (IHC) and found that the total fluorescence efficiency (TFE) of LYVE-1 staining from the inflamed tissues decreased by ~82.3% compared to the controls (Fig. 5D). This substantial down-regulation of LYVE-1 expression in LECs was purportedly caused by internalization and degradation of LYVE-1 receptors within lysosomes, which is induced by tumor necrosis factors TNFα and TNFβ during the acute inflammatory response^21^.

We next conducted *in vivo* LYVE-1 imaging of vehicles- and OXA-treated ears with µB enhanced molecular OCT at 48 h post-treatment (Fig. 5E and F). No contrast agent signals were observed in the lymphatics of either vehicle- and OXA-treated mice ear prior to injection. Comparable levels of contrast agent signals were observed in both ears at 5 min post-injection (Supplementary Fig. S8). At 1.5 h post-injection, however, we observed notably higher levels of contrast agents in vehicle-treated ears compared to ones challenged by OXA (Fig. 5E). Transverse images revealed that the contrast agent signals exhibited punctate patterns along the lymphatic endothelium in the pinnae of control ears while no contrast agent signals were observed in the inflamed pinnae after OXA challenge (Fig. 5F). The CLR of OXA-treated ears was 79.1% lower than that of the control ears (Fig. 5G, *P* = 0.00091). This observation is consistent with the results from *ex vivo* IHC characterization. The substantially reduced CLR could be explained by the significant down-regulation of LYVE-1 on the LECs in the CHS induced tissue of inflammation. After that, we used OCT to investigate the lymphatic vessel area density (VAD), a key indicator of skin inflammation^19, 22^. Two days after the treatment, the VAD of OXA-treated mice pinnae was 2.4 fold higher than that of the vehicle-treated counterpart (Fig. 5H), possibly due to lymphangiogenesis as well as enlargement of pre-existing lymphatic vasculature^22^. This observation further validated the acute inflammation state of the skin at the time of *in vivo* imaging.

## Discussion

Traditionally, lymphatic biomarkers are imaged by microscopy using fluorescent dyes as contrast agents^23–25^. However, fluorescent imaging techniques provide negligible structural and anatomical details for whole tissues. To circumvent this limitation, our lab recently demonstrated the use of contrast-enhanced OCT for improved high-resolution mapping of angiogenic tumor microvasculature and lymphatic function^26, 27^. In this report, we have extended the concept of contrast-enhanced OCT to enable molecular studies of lymphatic biomarker expression in healthy and inflamed murine models for the first time. This OCT-based platform offers an advantage over conventional fluorescence microscopy for *in vivo* studies of inflammation and other immune processes because of its enhanced imaging depth; this advantage is especially relevant for models of inflammation, in which swelling increases tissue thickness at the scale of hundreds of microns to millimeters. Furthermore, molecular contrast OCT enables targeted biomarker imaging over relatively large fields of view (4 mm × 4 mm) with ultrahigh contrast agent sensitivity and detection specificity. The use of pre-targeting strategy and a novel image processing algorithm offered unique advantages for simultaneous imaging of anatomical information and molecular functional details in living subjects. Summarily, this combinatorial approach has enabled the first *in vivo* targeted molecular imaging study with contrast-enhanced OCT.

The growth of lymphatic vessels (lymphangiogenesis) is actively involved in a number of pathological processes including tissue inflammation and tumor dissemination via metastasis. Notably, lymphangiogenesis is impaired in patients suffering from lymphedema, a debilitating condition characterized by the build-up of interstitial fluids (chronic tissue edema) and compromised immune surveillance functions. Molecular imaging of lymphatic biomarkers could be implemented to explore the molecular mechanisms governing lymphangiogenesis and tumor metastasis and provide new possibilities to treat these diseases. Thus, the functional role of LYVE-1 in lymphatic vessels, inflammation, and tumor lymphangiogenesis are vital areas of pre-clinical investigation. We used molecular OCT to demonstrate that LYVE-1 is down-regulated *in vivo* in models of acute CHS, which is consistent with previous findings obtained with *ex vivo* fluorescence labeling techniques^21^.

One limitation of the presented technique is associated with the molecular sensitivity. Because the µB contrast agent is ~1 µm in size, precise quantification of LYVE-1 receptor expression may be impeded. This challenge is not unique to molecular OCT; similar limitation exists for microbubble-based ultrasound imaging contrast agents. Additionally, labeling µBs to vascular biomarkers requires a pre-targeting approach, which seems to be a sophisticated, albeit necessary, method to deliver the contrast agents. Experiments in which anti-LYVE-1 conjugated µBs were directly injected in a single step did not achieve any targeting (Supplementary Fig. S9). It is conceivable that the targeting of large particles to cell-surface molecules can be obstructed by the strong drag force imparted to the particle by lymphatic fluid. The double-step targeting strategy overcame this targeting challenge by utilizing avidity effects as well as the high binding affinity of streptavidin for biotin (K_d_ = 10^−14^ to 10^−15^ M), which is several orders of magnitude higher than the affinity of anti-LYVE-1 antibody for LYVE-1 (K_d_ = 10^−7^ to 10^−9^ M).

OCT imaging instruments span a large range of spatial resolution and imaging speeds. While the system used in present research has intermediate resolutions of 5.7 µm laterally and 6.5 µm axially, and a moderate scan speed of 92 kHz, the method reported herein could be implemented for OCT systems operating at higher resolution and speed to allow more detailed visualization of the lymphatic molecular profiles with improved temporal resolution. If adopted, molecular OCT may enable clinical imaging of multiple molecular biomarkers across large lymphatic networks with sub-lymphangion resolution. Therefore we expect this method will provide novel insights and clinical research opportunities with respect to a variety of lymphatic-related diseases.

## Materials and Methods

### Chemicals and reagents

98% sulfuric acid (H_2_SO_4_) and 30% hydrogen peroxide (H_2_O_2_) were purchased from Fisher Scientific. (3-Aminopropyl)triethoxysilane (APTES), 4-ethoxymethylene-2 phenyl-2-oxazoline-5-one (oxazolone) and biotin were obtained from Sigma-Aldrich. (1-ethyl-3-(3-dimethylaminopropyl)carbodiimide hydrochloride) (EDC) and N-Hydroxysuccinimide (NHS) were purchased from Tokyo Chemical Industry (TCI) Co., Ltd and Acros Organics, respectively. Streptavidin coated polystyrene beads (1.09 μm in diameter) were purchased from Spherotech Inc. biotinylated LYVE-1 monoclonal antibody (Biotin-AntiLyve1) and biotinylated mouse IgG Isotype control (Biotin-Isotype) were purchased from eBioscience. Phosphate buffered saline (1X, pH 7.4) was obtained from Thermo Scientific Inc.

### Animals, instrumentation and image acquisition

All animal work was conducted in compliance with the guidelines of Stanford University Stanford University Animal Studies Committee for the Care and Use of Research Animals and the experimental protocols were approved by Stanford University’s Animal Studies Committee (APLAC protocol 27499). 6~8 weeks old female BALB/c mice (Charles River Inc., Wilmington, MA) were used for all imaging experiments. The hair on the ears was removed with Nair^TM^ hair removal cream prior to imaging. During imaging experiments, the mice were anesthetized by 2% isoflurane and placed on a 37 °C heating pad. The ear pinnae of the mice were attached to a mount by double-sided tape to minimize tissue motion. A layer of ultrasound gel was placed on top of the ear to optimize light transmission, and an anti-reflective glass was placed on the ultrasound gel to minimize the reflection at the air/glass interface. All images were acquired using a Telesto^TM^ spectral domain (SD) OCT system (ThorLabs, Newton, NJ) with a superluminescent diode (SLD) light source operated at 92 kHz. The SLD has a center wavelength of 1325 nm with the full bandwidth of 150 nm (∆λ = 1250~1400 nm), which provides an axial resolution of 6.5 µm. All images were acquired with a LSM02 lens (ThorLabs, Newton, NJ), providing a lateral resolution of 5.7 µm (FWHM) and a depth of view (DOV) of 53 µm in water. Each B-scan consists of 357 A-lines covering a range of 2 mm on the sample. Each 3D data volume consists of 357 consecutive B-scans with 8 B-scan averages for each lateral position. In order to cover a large field of view (4 mm × 4 mm) of the mouse ear, 2 × 2 adjacent 3D scans were acquired. The acquired 3D data volumes were then stitched together with post-processing algorithms.

### Design of phantom experiment

Glass capillary tubes (0.4 mm inner diameter and 75 mm in length) were obtained from Diamond Scientific Co. (Broomall, PA). The biotinylation process for functionalizing glass capillary tube inner surfaces is shown in Supplementary Fig. S10. First, the inner surface capillary tube was treated with piranha solution (mixture of 98% H_2_SO_4_ and 30% H_2_O_2_ in a ratio of 4:1) for 15 min to generate hydroxyl groups on the glass surface. The capillary tube was then thoroughly washed with water and ethanol followed by incubation in ethanol solution with 10% APTES for 2 h, resulting in the formation of an amino-terminated, self-assembled silane monolayer (SAM) on the glass surface. The silanized capillary tube was then immersed in an ethanol solution containing 0.1 M EDC, 0.02 M NHS and 1 mM biotin for 2 h at room temperature to conjugate the biotin molecules onto capillary tube glass surface. Capillary tubes without surface modification were used as controls. All capillary tubes were mounted to a stage with double-sided tape. In a typical experiment, one end of the given capillary tube was connected to a catheter attached to a syringe. 0.1 nM µBs were continuously injected into the capillary tube using a syringe pump operating at a speed of 0.1 mL min^−1^. Pre-wash B-scan OCT images were acquired while beads were flowing through the capillary tube. Next, the capillary tube was washed by injecting DI water by the syringe pump at a speed of 0.1 mL min^−1^ for 30 min until no free flowing beads could be observed. Post-wash B-scan OCT images were acquired after the all free flowing beads had been cleared from the capillary tube.

### Longitudinal imaging of LYVE-1 targeted µBs *in vivo*

The ears of pre-targeted and non-targeted groups of mice were injected s.c. with 5 µL of biotinylated anti-LYVE-1 and biotinylated isotype control antibodies, respectively. For competitive assay group, the mice ears were injected with a 5 µL mixture of non-biotinylated anti-LYVE1 and biotinylated anti-LYVE1 at a ratio of 10:1. 15 min after the injection, 3D OCT images covering a FOV of 4 mm × 4 mm × 2 mm on the ear were acquired. After 30 min of injecting the antibodies, a total 10 µL of 2 nM stp-µB was administered s.c. at two different locations ~1 mm apart from antibody injection site on the ear. 3D OCT images of the same FOV were acquired longitudinally at 5 min, 15 min, 30 min, 60 min, 90 min and 120 min after the µB injection.

### *In vivo* LYVE-1 imaging in murine model of acute CHS

6–10 week old BALB/c female mice were sensitized by topical application of 50 µL of 2% (w/v) oxazolone (in acetone/olive oil, 4:1 vol/vol) on their shaved abdomen. After 5 days of the sensitization (day 0), the dorsal surfaces of the left ears were topically challenged with 10 µL of 1% (w/v) oxazolone (in acetone/corn oil, 4:1 vol/vol), while the right ears (control) were treated with 10 µL of the vehicle solution (acetone/corn oil, 4:1 vol/vol) alone. The thickness of the left and right ears was measured with a caliper every day after the challenge (starting from day 0 until day 3). *In vivo* OCT imaging of LYVE-1 was conducted on day 2 post-challenge. Left and right ear biopsies (n = 4 from each group) were collected 2 days after the challenge for histological and immunohistochemical assessment.

### Post-image processing

The phase variance signals were calculated from 8 consecutive B-scans acquired at the same transverse location within 30 milliseconds. This acquisition allows visualization of all movements of light-scattering particles including red blood cells flowing in blood vessels^5, 28, 29^ and the s.c. injected µBs, which were drained to the lymphatic vessels. Lymph vessels were segmented using an automatic algorithm based on the fact that lymph fluid is optically transparent and therefore the lymphatic vessels exhibit very low OCT signals. The signals of µBs (lymphatic contrast signals) were segmented by gating the phase variance signals with the binary mask of lymphatic vessels. Blood vessel maps (angiograms) were then obtained by subtracting the lymphatic contrast signals from phase variance signals (detailed steps of this process are presented in the Supplementary Mateirals).

### Histology and immunohistochemistry

Isolated tissues were immersion-fixed in 10% paraformaldehyde for 4 hours and rinsed in 70% ethanol overnight. The tissue samples were then embedded in paraffin, and slides were prepared with 5 µm sections for hematoxylin-and-eosin (H&E) staining and immunohistochemistry (IHC). For IHC, the tissue sections were de-paraffinized and antigen retrieval was performed in citric acid buffer for 1 h at 80 °C. Sections were then blocked in 10% Bovine Albumin Serum (BSA) for 1 h at room temperature and incubated with primary anti-LYVE-1 antibody (eBioscience) overnight at 4 °C. Slides were rinsed in PBS-Triton X-100 (0.3% v/v) followed by staining with Alexa 594-conjugated secondary antibody (Abcam, ab150168) for 1 h. After washing, the tissue sections were counterstained with DAPI and mounted in Vectashield medium (H-1000; vector Laboratories, Inc., Burlingame, CA). For whole-mount staining, the tissues were first fixed in 4% paraformaldehyde for 4 h at room temperature, blocked in PBS-Triton X-100 (0.3% v/v) supplemented with 3% BSA (Bovine Albumin Serum) for 48 hours at 4 °C. Then they were incubated with primary antibodies for 5 days at 4 °C. After washing, the samples were incubated with secondary antibodies for 48 h, and counterstained with DAPI for 30 min. Slides were then mounted in Vectashield medium and imaged using a 2-photon confocal microscope (Prairie Technologies Ultima IV).

### Statistics

Statistical analysis for *in vitro* and *in vivo* experiments was performed with GraphPad Prism (GraphPad Software). A two-tailed Student’s unpaired *t* tests were applied to compare the targeted versus non-targeted and control versus treated groups of mice. The significant levels were set at *P* < 0.05 (*) and *P* < 0.001 (**). Independent experiments were conducted with a minimum of four biological replicates per condition. Error bars represent SEM.

## Electronic supplementary material

Supplementary Materials

Supplementary Movie S1

Supplementary Movie S2

Supplementary Movie S3
