# Supplementary material for: In Vivo Molecular Optical Coherence Tomography of Lymphatic Vessel Endothelial Hyaluronan Receptors
Source: Sci Rep. 2017 Apr 24;7:1086. doi: 10.1038/s41598-017-01172-x (PMC5430649; doi:10.1038/s41598-017-01172-x)
Supplement: Supplementary file 1 — Supplementary Materials [file 41598_2017_1172_MOESM1_ESM.pdf]

Supplementary Materials for

In Vivo Molecular Optical Coherence Tomography of Lymphatic Vessel Endothelial Hyaluronan Receptors

Peng Si[1,2], Debasish Sen[1,2], Rebecca Dutta[1,2], Siavash Yousefi[1,3], Roopa Dalal,[4] Yonatan Winetraub,[1,2,6] Orly Liba[1,2,5,6] and Adam de la Zerda[1,2,5,6]*

[1]Molecular Imaging Program at Stanford, [2]Department of Structural Biology, [3]Department of Radiation Oncology, [4]Department of Ophthalmology, [5]Department of Electrical Engineering, [6]Bio-X Program, Stanford University, 299 Campus Drive, Stanford, California 94305

Supplementary methods

Phase variance algorithm

Phase variance was calculated using Matlab® (Mathworks, Natick, MA). The phase variance of each transverse plane of a 3D OCT image was calculated from a set of 357 A-scans and 8 B-scans at the same location. The outline of the ear was detected and signals outside the ear tissue were removed using a threshold obtained as an average of the 8 B-scans. The phase φ_{zij} of a pixel is given by $\angle C_{zij}$ where C_{zij} is the complex OCT signal at depth position *z* of the ith A-scan and j^{th} B-scan. A set of 30 pixels from the top outline of ear in the $(i-1)^{th}$, i^{th} and $(i+1)^{th}$ A-scans of jth B-scan were then used to calculate the background phase $\varphi_{ref}(i,j)$ for i^{th} A-scan in j^{th} B-scan. Changes in phase between adjacent B-scans for each pixel were then calculated by the equation below:

$$\Delta\varphi_{zij} = \angle(C_{zij}/C_{zij+1}) - \varphi_{ref}(i,j) \qquad (1)$$

The phase variance of 8 consecutive B-scans within one BM-scan is calculated as:

$$\sigma^2_{\Delta\varphi} = \frac{1}{7} \times \sum_{j=1}^{7} \left[\Delta\varphi_{zij} - \frac{1}{7} \times \sum_{j=1}^{7} \Delta\varphi_{zij}\right]^2 \qquad (2)$$

Segmentation of lymphatic vessels

The lymphatic vessels were segmented with an algorithm based on Hessian filters.[7,8] This process is briefly explained herein. The local behavior of an image $\mathrm{I}(\mathrm{x}, \mathrm{s})$ at scale s and location x can be expressed by its Taylor series expansion up to the second order given by:

$$I(x + \delta x, s) \approx I(x, s) + \delta x^T \nabla(I)_s + \delta x^T H(I)_s \delta x \tag{3}$$

where $\nabla(.)_s$ and $H(.)_s$ are the gradient vector and Hessian matrix of the image at scale s, respectively. Since our volume cross-section images are discrete signals, finding their 2-D first-order and second-order derivative can be ill-posed. Using the concepts of linear scale space theory, differentiation can be defined as a convolution with derivatives of a Gaussian:

$$\frac{\partial}{\partial x} I(x, s) = s^{\gamma} I(x, s) * \frac{\partial}{\partial x} G(x, s) \tag{4}$$

where

$$G(x, s) = \frac{1}{\sqrt{2\pi s^2}} e^{-\frac{||x||^2}{2s^2}} \tag{5}$$

γ is the derivative normalization parameter and $||.||$ is the Euclidean norm operation. The second order derivative can be expressed as:

$$\delta x^T H(I)_s \delta x = \left(\frac{\partial}{\partial x}\right)\left(\frac{\partial}{\partial x}\right) I(x, s) = s^{2\gamma} I(x, s) * \frac{\partial^2}{\partial x^2} G(x, s). \tag{6}$$

By analyzing the eigenvalues and eigenvectors of the Hessian matrix, the principal direction of the local structure can be extracted which is the direction of the smallest curvature (along the vessel). For an ideal tubular three-dimensional structure, the relationship between eigenvalues of the Hessian matrix is given by:

$$|\lambda_3| \approx 0,\ |\lambda_3| \ll |\lambda_2|,\ \lambda_2 \approx \lambda_1 \text{ and } (|\lambda_3| \leq |\lambda_2| \leq |\lambda_1|). \tag{7}$$

Based on the second order ellipsoid, three geometric ratios are defined as:

$$R_A = \frac{|\lambda_2|}{|\lambda_3|},\ R_B = \frac{|\lambda_1|}{\sqrt{\lambda_2 \lambda_3}} \text{ and } R_C = \left||H|\right|_F = \sqrt{\textstyle\sum_{j \leq D} \lambda_j^2}. \tag{8}$$

where R_A refers to the largest cross-section area of the ellipsoid that can distinguish between plate-like and line-like structures. R_B accounts for the deviation from a blob-like structure and R_C is the Frobenius matrix that can distinguish background

noise where no scattering structures is present. The “vessel-ness” function at scale s is defined as:

$$v_0(s) = \begin{cases} 0 & if\ \lambda_2 > 0\ or\ \lambda_3 > 0 \\ \left(1 - e^{-\frac{R_A^2}{2\alpha^2}}\right) * e^{-\frac{R_B^2}{2\beta^2}} * (1 - e^{-\frac{R_C^2}{2\theta^2}}) \end{cases} \quad (9)$$

where α, β and θ are thresholds which control the sensitivity of the line filter to the measures R_A, R_B and R_C. The idea behind this expression is to map the features into probability-like estimates of vessel-ness according to different criteria.

The vessel-ness measure is analyzed at different scales. The response of the line filter will be maximum at a scale that approximately matches the vessel size.

$$V_0(\gamma) = \arg max_s \ v_0(s,\gamma),\ s_{min} < s < s_{max} \quad (10)$$

where s_{min} and s_{max} are lower and upper bound in the range of scale (vessel sizes).

Image stitching and registration

Four adjacent 3D images with FOV of 2 mm x 2 mm were stitched to one 3D image with a large FOV of 4 mm x 4 mm using Matlab®. The stitched phase variance images were used to align the OCT images acquired at different time post-μB injection. The underlying assumption was that major blood vessels (1) do not move much between time points and (2) have a significantly higher phase variance signal than the μBs, and therefore are distinguishable from background. The stitched phase variance image at each post-μB injection time point is presented by a 3D matrix: $V(t) = V(x,y,z;t)$, where z is the depth position, x is the lateral location along the B scan direction, y is the complementary and t is the time post-μB injection.

The next step is to align all post-injection phase variance matrixes $V(t)$ against the

post-120 min injection phase variance matrix$V(t=120)$. Alignment was done to account for global translations $(\Delta x, \Delta y, \Delta z)$ and rotations along z axis: α

Estimation of $\Delta x, \Delta y, \Delta z, \alpha$ was done in the following steps. First, a MIP image $V^m(x,y;t)$ was calculated for both $V(t)$ and $V(t=120)$:

$$V^m(x,y;t) = \max_z \left[V(x,y,z;t) \cdot \begin{cases} 1 \; if \; V(x,y,z;t) > th \\ 0 \; otherwise \end{cases}\right] \quad (11)$$

where threshold value th was selected such that the majority of max projection image is originated from major blood vessels phase variance. Then, exhaustive search was used to minimize the mismatch between V(t) and V (120):

$$\min_{\Delta x, \Delta y} \sum_{x,y} [V^m(x+\Delta x, y+\Delta y; t) - V^m(x,y;t=120)]^2 \quad (12)$$

Then, Matlab's optimization toolbox was use to optimize the following expression:

$$\min_{\Delta x, \Delta y, \alpha} \sum_{x,y} [V^m(x \cdot \cos\alpha - y \cdot \sin\alpha + \Delta x, x \cdot \sin\alpha + y \cdot \cos\alpha + \Delta y; t) - V^m(x,y;t=120)]^2 \quad (13)$$

Initial guess of $\Delta x, \Delta y$ was taken from previous step and initial guess of $\alpha = 0$ Finally, in order to estimate Δz, 3 B-scans were used: $y_1 = \frac{S_y}{4}, y_2 = \frac{S_y}{2}, y_3 = \frac{3S_y}{4}$, where S_y is the number of B scans in $V(t)$. The following expression was used to further the exhaustive search:

$$\min_{\Delta z} \sum_{x,z} \sum_{y \in (y_1,y_2,y_3)} [V(x \cdot \cos\alpha - y \cdot \sin\alpha + \Delta x, x \cdot \sin\alpha + y \cdot \cos\alpha + \Delta y, z + \Delta z; t) - V(x,y,z;t=120)]^2 \quad (14)$$

where $\Delta x, \Delta y, \alpha$ were constants taken from previous step. Finally, all post-μB injection OCT images were aligned with the 120 min post-μB injection OCT image using the calculated parameters $\Delta x, \Delta y, \Delta z, \alpha$.

Segmentation of lymphatic contrast signals and blood vessels

The PV signal of μBs are obtained by gating the phase variance signals of a 3D OCT image by the 3D lymph vessel mask segmented from the 120 min post-μB injection OCT image. The image of blood vessels is obtained by subtracting the PV signal of μBs from the phase variance signals in 3D images. The maximum intensity projection (MIP) image of blood vessels, lymph vessels and μBs were created by superimposing the stitched 3D images of segmented blood vessels, lymphatic vessels and μBs which are pseudo-colored with red, blue, and green respectively, followed by projecting the pixels with the highest intensity in all enface frames to a 2D image.

Supplementary figures

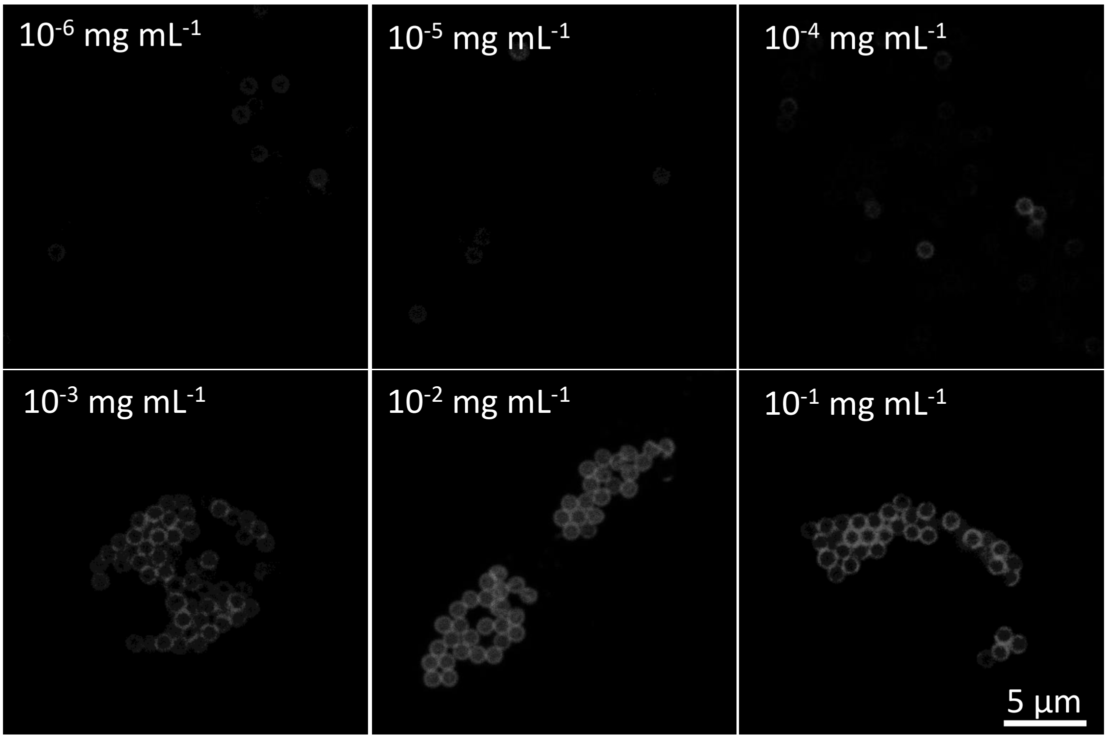

Supplementary Fig. S1. Confocal fluorescent images showing the center planes of FITC-conjugated μBs, the resulting products of a titration assay in which the biotin-FITC concentration ranges from 10^{-6} mg mL^{-1} (upper left) to 10^{-1} mg mL^{-1} (bottom right).

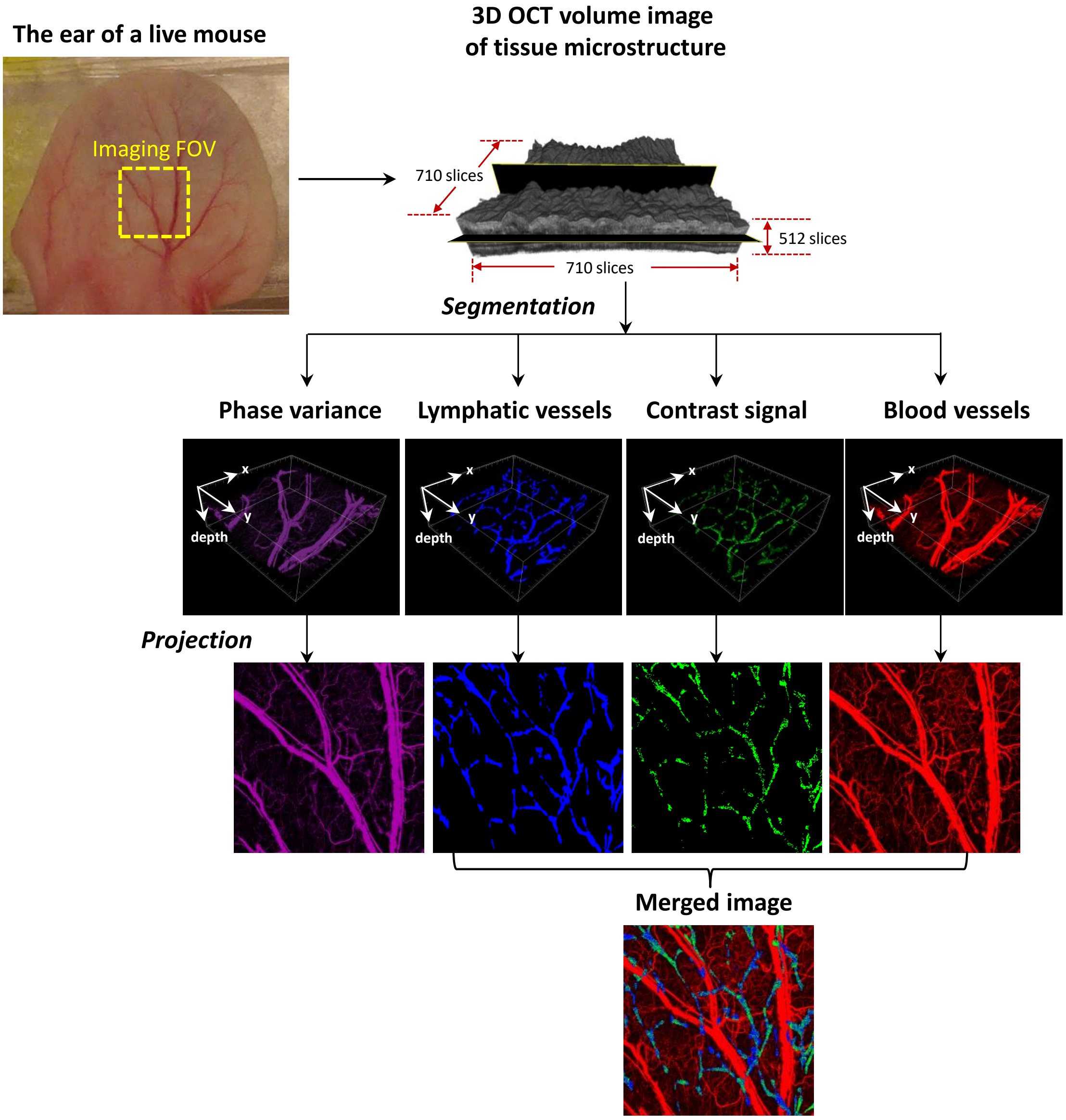

Supplementary Fig. S2. The workflow of obtaining *en face* merged projection images of angiogram, lymphangiograms, and lymphatic contrast signals by post-image processing. The phase variance and lymphatic vessels were segmented from 3D OCT volume image of tissue microstructure, then the lymphatic contrast signals were obtained by gating the phase variance signals by the lymphatic vessels, and the blood vessels were obtained by subtracting the lymphatic contrast signals from the original phase variance image. The 2D *en face* images were obtained by maximum intensity projection from the 3D images. Finally, the *en face* merged image was obtained by superimposing lymphatic contrast signals, lymphangiograms, and angiogram on top of each other.

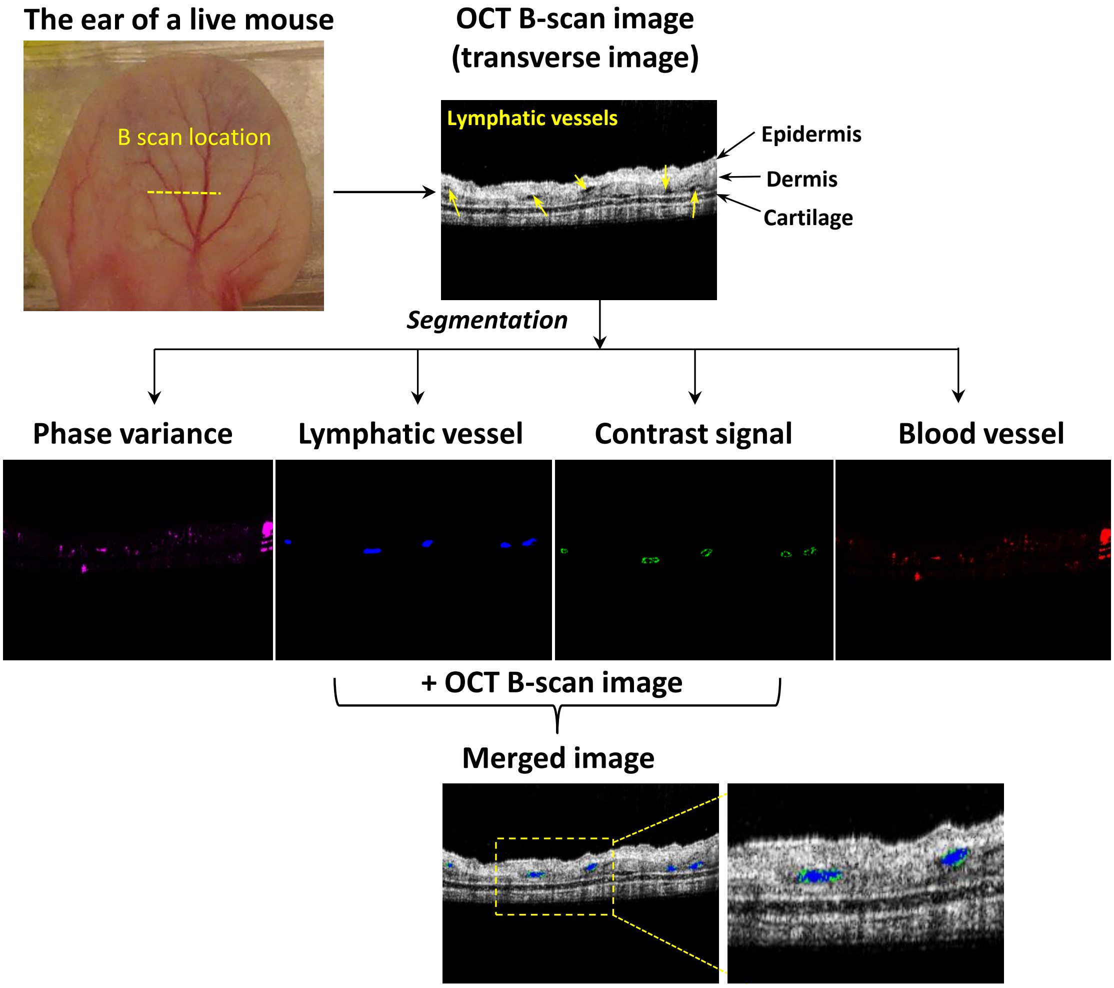

Supplementary Fig. S3. The workflow of obtaining transverse merged images of tissue microstructure, lymphatic vessels, and lymphatic contrast signals by post-image processing. The OCT B-scan image clearly reveals the anatomy of ear tissue including epidermis, dermis, cartilage, and lymphatic vessels, as labeled on the right image of the top panel. The phase variance and lymphatic vessels can be segmented directly from the OCT B-scan image. Then the lymphatic contrast signals were obtained by gating the phase variance signals by the lymphatic vessels, and the blood vessels were obtained by subtracting the lymphatic contrast signals from the original phase variance image. The final merged transverse image was obtained by overlaying the transverse images of lymphatic contrast signals, lymphatic vessels, and blood vessels on top of each other.

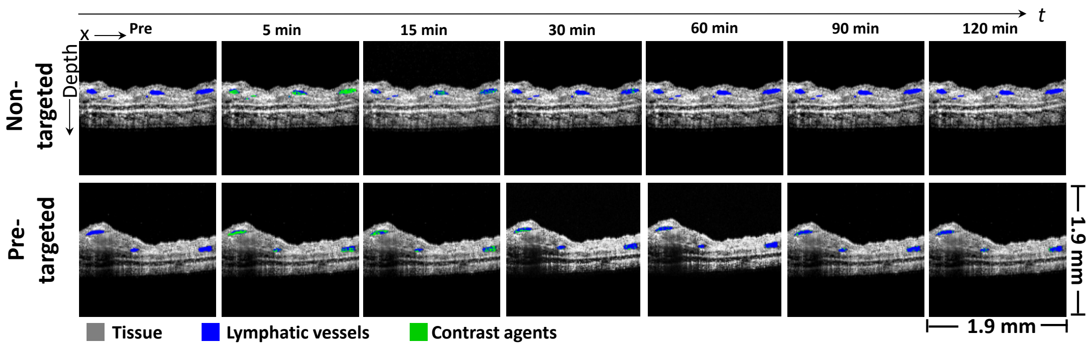

Supplementary Fig. S4. The large FOV transverse images showing OCT microstructure, lymphatic vessels and lymphatic contrast signals of non-targeted and pre-targeted mice ears at different longitudinal imaging time points.

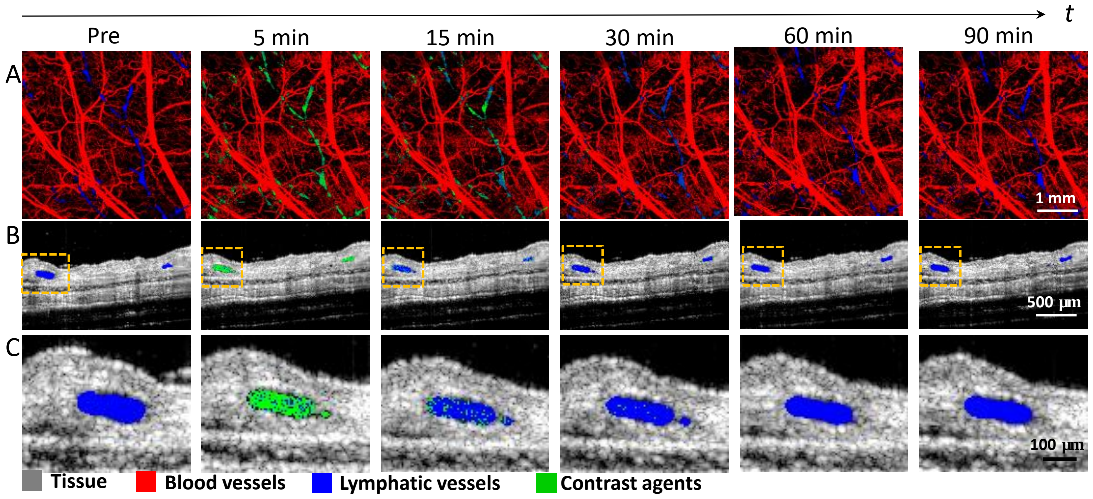

Supplementary Fig. S5. The time-lapse *en face* (A), transverse (B) and enlarged transverse (C) molecular OCT images showing blood vessels (red), lymphatic vessels (blue), contrast agent signals (green) and tissue microstructure (gray) of a mouse ear subjected to competitive assay at different longitudinal imaging time points.

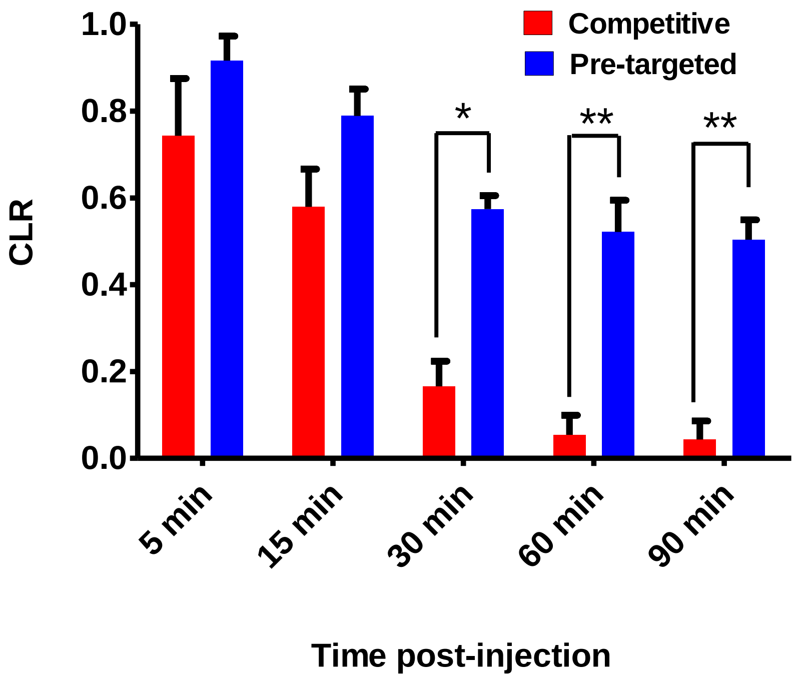

Supplementary Fig. S6. The contrast-to-lymphatic ratios (CLR) of the competitive assay versus the pre-targeted groups of mice at different imaging time points post-injection. Error bars are ±SEM of the mean (n=4 mice). *P<0.05, **P<0.001.

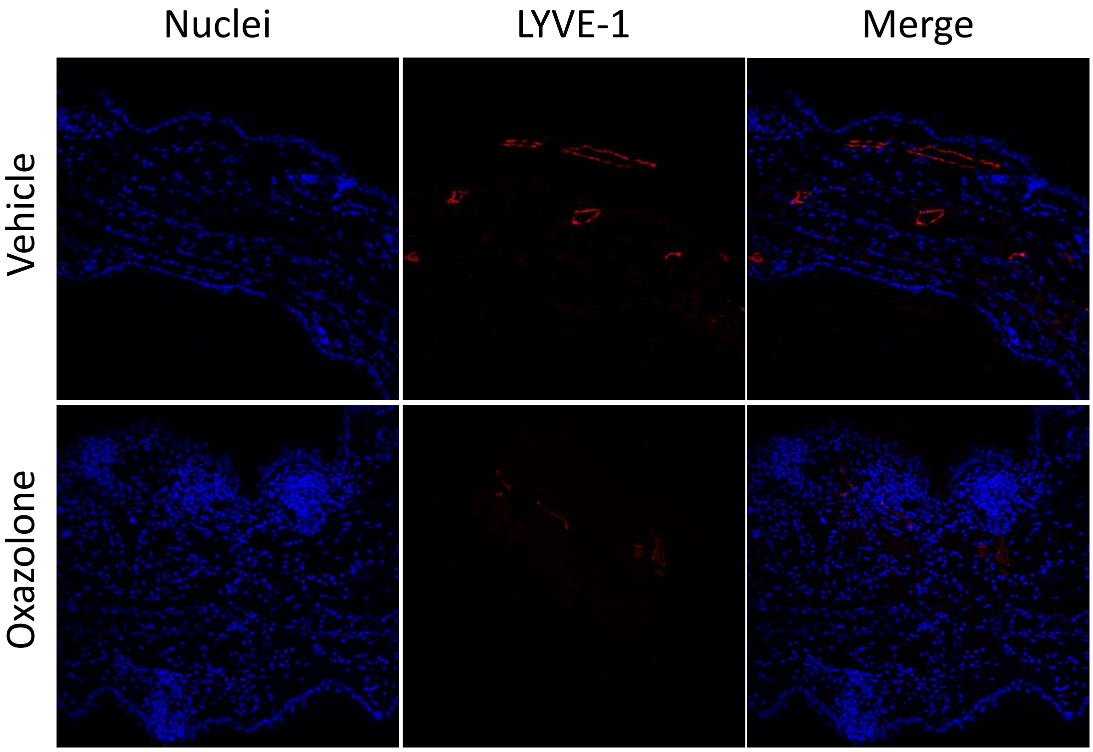

Supplementary Fig. S7. The emissions from DAPI and Alexa 594 staining on the vehicle- and OXA-treated tissue sections after 2 days of challenge. Blue: the DAPI nuclear staining. Red: LYVE-1 staining by rat-anti-mouse LYVE-1 antibody (primary) and goat-anti-rat antibody conjugated with Alexa 594 (secondary).

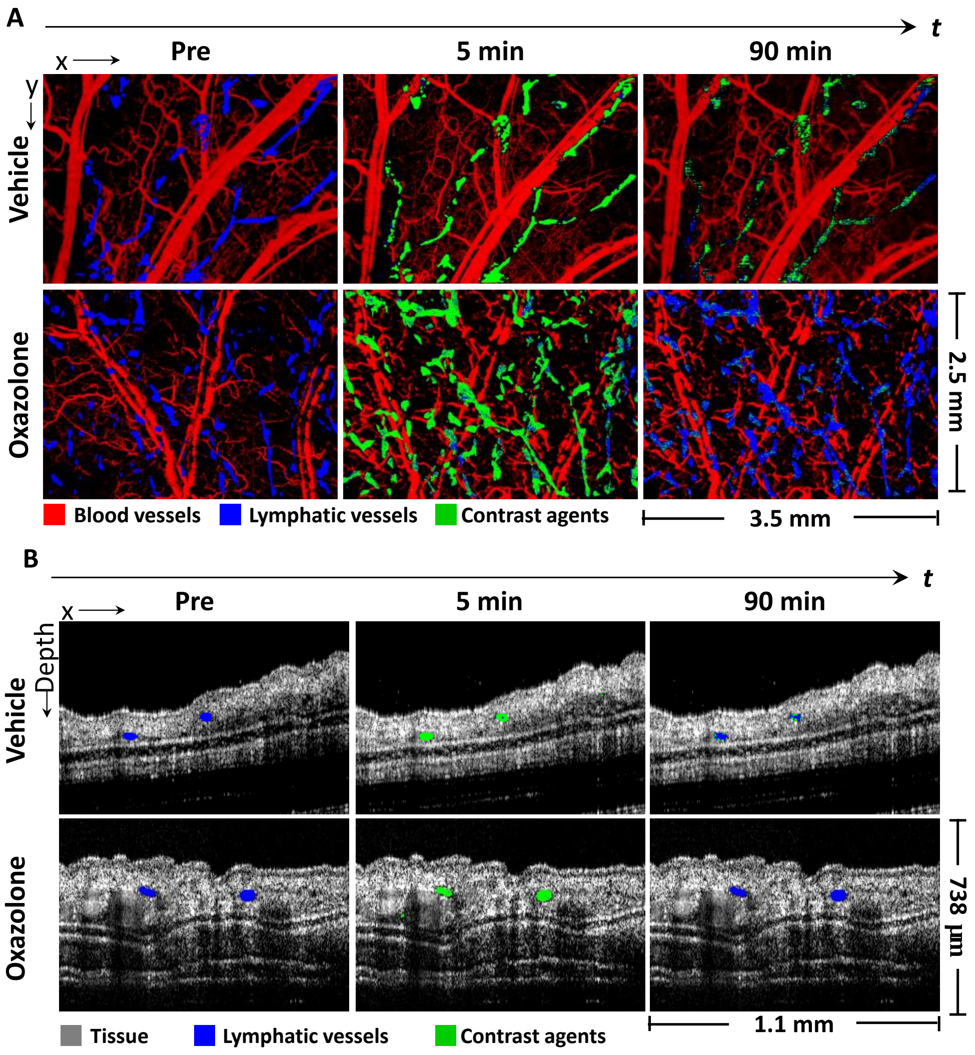

Supplementary Fig. S8. The *en face* (A) and transverse (B) merged images of control and inflamed mice ears at the time points of pre-injection, 5 min, and 90 min post-injections.

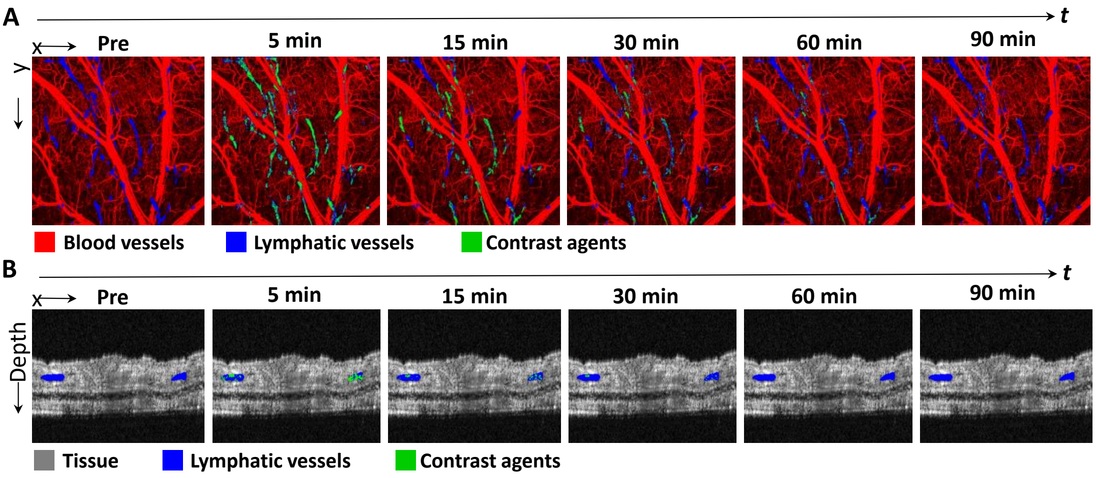

Supplementary Fig. S9. The time-lapse *en face* (A) and transverse (B) merged images of the mouse ear tissue before and at different time points after injecting the AntiLyve1 conjugated µBs.

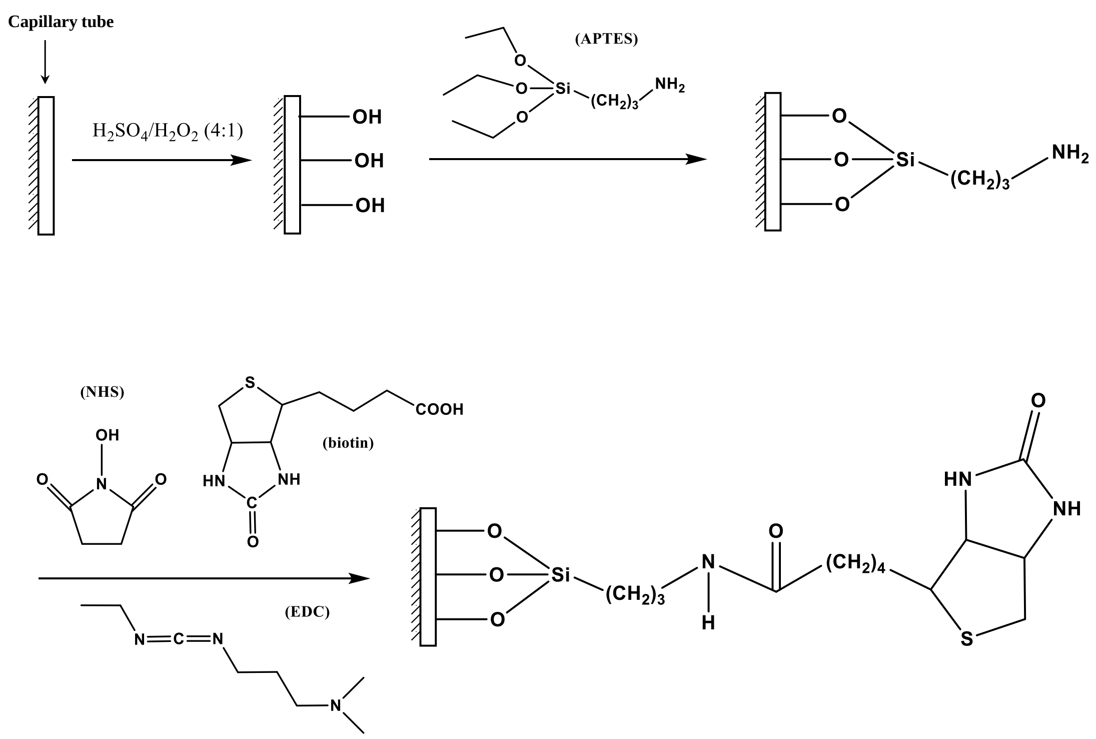

Supplementary Fig S10. Schematic illustration showing the surface modification steps of the capillary tube inner surface with biotin molecules.

Supplementary movies

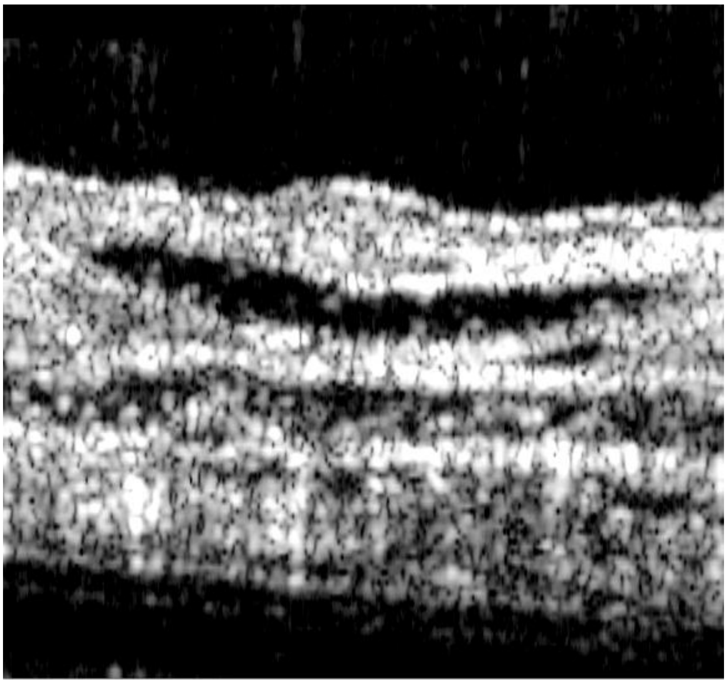

Supplementary Movie S1. Time-lapse OCT recording of a capillary lymphatic vessel in a pre-targeted mouse ear before and after injecting stp-µBs.

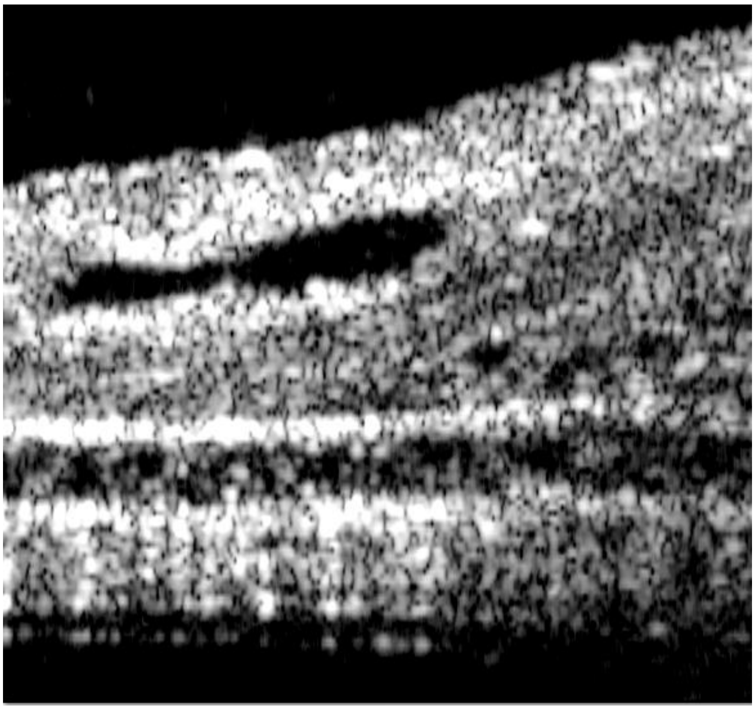

Supplementary Movie S2. Time-lapse OCT recording of a capillary lymphatic vessel in a non-targeted mouse ear before and after injecting stp-µBs.

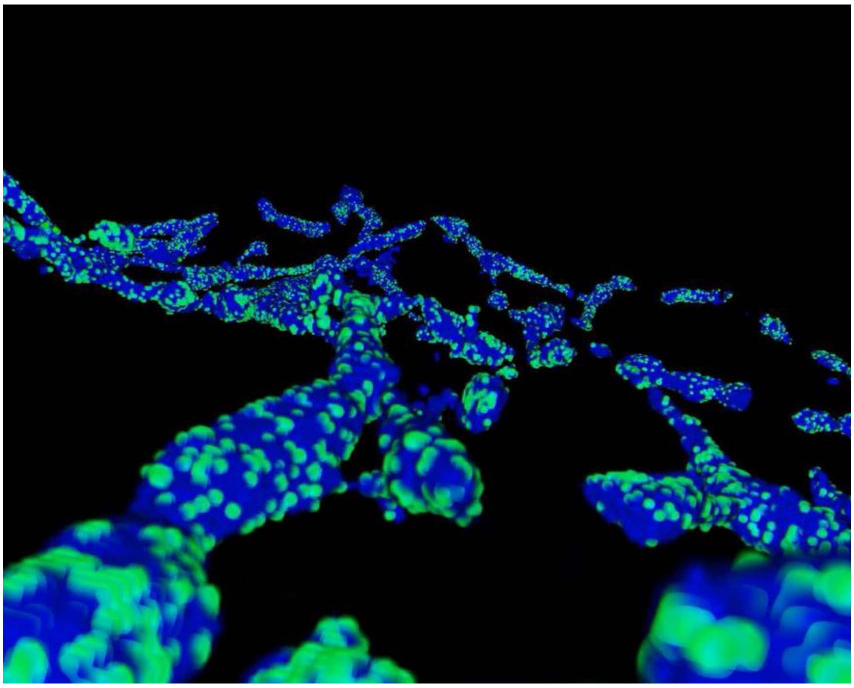

Supplementary Movie S3. 3D rendering OCT image of the lymphatic vessels and LYVE-1 targeted µB at 90 min post-injection.
